# The UK Burden of Injury Study – a protocol. [National Research Register number: M0044160889]

**DOI:** 10.1186/1471-2458-7-317

**Published:** 2007-11-08

**Authors:** Ronan A Lyons, Elizabeth E Towner, Denise Kendrick, Nicola Christie, Sinead Brophy, Ceri J Phillips, Carol Coupland, Rebecca Carter, Lindsay Groom, Judith Sleney, Phillip Adrian Evans, Ian Pallister, Frank Coffey

**Affiliations:** 1 School of Medicine, Swansea University, Swansea. SA2 8PP, UK; 2 Centre for Child and Adolescent Health, University of West England, Bristol. BS6 6JS, UK; 3 Division of Primary Care, School of Community Health Sciences, Institute of Clinical Research, Nottingham University, NG7 2RD, UK; 4 Surrey Injury Research Group, Postgraduate Medical School, University of Surrey, Daphne Jackson Road, Manor Park, Guildford, Surrey, GU2 7WG, UK; 5 Emergency Department, Morriston Hospital, Heol Maes Eglwys, Morriston, Swansea SA6 6NL, UK; 6 Emergency Department, Nottingham University Hospitals, NHS Trust, Queen's Medical Centre Campus, Derby Road, Nottingham NG7 2 UH, UK

## Abstract

**Background:**

Globally and nationally large numbers of people are injured each year, yet there is little information on the impact of these injuries on people's lives, on society and on health and social care services. Measurement of the burden of injuries is needed at a global, national and regional level to be able to inform injured people of the likely duration of impairment; to guide policy makers in investing in preventative measures; to facilitate the evaluation and cost effectiveness of interventions and to contribute to international efforts to more accurately assess the global burden of injuries.

**Methods/Design:**

A prospective, longitudinal multi-centre study of 1333 injured individuals, atttending Emergency Departments or admitted to hospital in four UK areas: Swansea, Surrey, Bristol and Nottingham. Specified quotas of patients with defined injuries covering the whole spectrum will be recruited. Participants (or a proxy) will complete a baseline questionnaire regarding their injury and pre-injury quality of life. Follow up occurs at 1, 4, and 12 months post injury or until return to normal function within 12 months, with measures of health service utilisation, impairment, disability, and health related quality of life. National estimates of the burden of injuries will be calculated by extrapolation from the sample population to national and regional computerised hospital in-patient, emergency department and mortality data.

**Discussion:**

This study will provide more detailed data on the national burden of injuries than has previously been available in any country and will contribute to international collaborative efforts to more accurately assess the global burden of injuries. The results will be used to advise policy makers on prioritisation of preventive measures, support the evaluation of interventions, and provide guidance on the likely impact and degree of impairment and disability following specific injuries.

## Background

The burden of injury can be assessed from a number of perspectives; that of the individual, their family, communities, the health service, the economy and society as a whole. The Global Burden of Disease and Injuries Study (GBDI) by the World Health Organization has helped to establish international methods of measuring the burden of disease and injury on a global basis [1], while the notion of Disability Adjusted Life Years (DALYs [2]) was developed to broaden the measurements of the impact of disease and injury. However, the calculation of DALYs has been subject to a number of different approaches, and there is little evidence relating to their validity, reliability and sensitivity as a measurement instrument [3]. The GBDI study, whilst representing a marked improvement in our knowledge of the global impact of injuries, has major shortcomings, including a reliance on professionals estimates on the severity and duration of post injury disability rather than the collection of empirical data and an absence of data on several injury categories, particularly those which are less life threatening [1]. Whilst the GBDI approach has been adopted to measure the national burden of injuries in several countries and there are a number of burden of injury studies which have been carried out for specific types of injuries, [4,5] empirical prospectively collected data on comprehensive injury populations is extremely rare.

The most comprehensive study to date is a Dutch study which includes substantial numbers of patients but was somewhat limited by a low response rate and absence of pre-injury measurement of disability, due to recruitment by letter some 2 months post injury [6]. There has been much investigation into how routine measures collected in hospitals can provide indicators of severity. However, indicators such as hospital admission and length of stay may not be reliable measures of long-term consequences [7-10]. For example, Barker et al [7] demonstrated that between half and three quarters of injuries in children and young adults that resulted in permanent disability were treated as outpatients and that most of these resulted from hand injures. Even within the category of hand injuries, seemingly similar categories of anatomical injuries can have widely different consequences. Loss of a little finger had very limited functional consequences, whereas loss of thumb is so devastating that transplantation of a big toe is common practice to reduce the functional severity of the injury [11].

Langley and Cryer have demonstrated that trends in hospitalisation rates for all injuries and hospital lengths of stay are not reliable indicators of the incidence of serious injuries [8,9]. A recently published review of post injury disability studies provided recommended guidelines on the selection of patients and measurement instruments and the timing of their application in future burden of injury studies. These guidelines proposed an integrated approach whereby [10] complementary measures of health related quality of life (HUI3 and EQ-5D), time off work and restricted activities are collected simultaneously in order to study the inter-relationship between variables and more comprehensively describe the burden of injury in patients attending emergency departments or admitted to hospital. In addition, a 'pre-injury' quality of life measure is also recommended which can only realistically be assessed shortly after injury. This recommendation is supported by recently published research which shows that pre-injury quality of life scores in injured individuals differ significantly from age and sex matched general population scores [12].

One of the authors of this study (RAL) was involved in the development of the guidelines which influenced the design of this study. In addition, measures of severity of threat to life (Abbreviated Injury Scale (AIS [13]) and International Classification of Disease-Based Severity Score (ICISS) [14]) have been included in this study to facilitate comparison with international studies using hospital separation data [14]. The combination of post injury disability data with injury mortality data is required to measure the total burden of injuries and provide comparison with studies using QALYS and DALYS [3,15]. This study also includes a qualitative component to complement the largely quantitative approach used in this and existing studies. This mixed methods approach is used as it is recognised that no instrument or collection of instruments comprehensively captures all aspects of the burden of injury on individuals and their families.

### Aims

The aim of the study is to provide estimates of the UK burden of injury in order to help policy makers and practitioners prioritise intervention measures and to contribute to international efforts to more accurately assess the global burden of injuries.

### Objectives

To measure the impact of varying severities of injuries for children and young people, adults and older people in relation to:

1. The effects on health related quality of life and disability.

2. The consequences for health and social care services in terms of resource utilisation.

3. The effects on the economy and the labour market in terms of working days and working life years lost.

4. The personal impact of injury to the individual and their experiences following the injury.

5. The total UK burden of injuries by combining study specific disability data with administrative health and mortality datasets.

## Methods/Design

### Participants

This is a mixed quantitative and qualitative prospective longitudinal multi-site study involving patients with a comprehensive spectrum of injuries. Four centres in the UK (Swansea, Nottingham, Bristol, Surrey) will recruit emergency department attendees and those admitted to hospital following an injury. Tables 1, 2 and 3 describe the stratified sample of patients to be recruited into the study. This stratification was designed to include the most common injuries (e.g. fractures, sprains), the potentially most disabling injuries (e.g. hand or eye injuries) and less common but important injuries (e.g. head injury, burns). Potential participants will be identified by emergency department staff. Those who agree to discuss the study with a member of the research team will have the study explained to them, be given the study information sheet and a consent form to complete. For younger children or adults who can not give consent themselves, a proxy (relative or carer) will be asked to read the information sheet and give assent to the study.

**Table 1:** Number of participants to be recruited per centre, by age group and injury type [Non admitted patients]

Injury type	Total number	Number in each age group		
		5–24	25–59	60+
Fracture/Dislocation*	200	64	68	68
Laceration	80	24	28	28
Bruises/Abrasions	80	24	28	28
Sprains	160	60	60	40
Burns/Scalds	60	20	20	20
Head injury	60	20	20	20
Eye injury	20	Any age		

*See table 2

**Table 2:** Break down of fractures/dislocations and sprains;

	Age group		
	5–24	25–59	60+
**Fractures/Dislocations**			
Wrist	12	12	12
Upper arm/elbow	12	12	12
Ankle	12	12	12
Digits	12	12	12
Others	16	16	16
**Sprains**			
Wrist	12	12	8
Ankle	12	12	8
Knee	12	12	8
Neck strain	12	12	12
Other	12	12	4

**Table 3:** Number of participants to be recruited per centre by age group and anatomical site of injury [Admitted patients or In-patients].

	Age group		
	5–24	25–59	60+
**Number**	**220**	**220**	**220**
Thermal (any site)	20	20	20
Head/face	40	40	20
Thorax	20	20	20
Abdomen/Pelvis	16	16	20
Hip	*	*	40
Leg	40	40	24
Arm	40	40	24
Wrist	16	16	20
Hand	16	16	16
Others including neck injuries	16	16	16

* Hip can be included in leg injuries in people aged < 60.

### Exclusion/Inclusion Criteria

Inclusion criteria: patients aged 5 years and over, with injury types as specified in tables 1, 2, 3, which occurred up to 2 weeks prior to the date of recruitment or within 4 weeks if the patient is admitted to hospital with a serious injury, who are able to give consent and complete questionnaires OR who have a suitable proxy who can assent to their participation and complete questionnaires in the future.

Exclusion criteria: patients with injury types not specified in tables 1 and 3, children below the age of 5, those who are unable to give consent themselves and do not have a suitable proxy that can assent to their participation and those who are unable to complete questionnaires in the future. Children less than 5 years have been excluded due to a lack of suitable measurement instruments. Patients with no address or those who are leaving the UK permanently and patients with stings and foreign bodies in the ear have been excluded.

### Measures

At baseline (day of recruitment into the study) participants will be asked to complete a questionnaire containing questions on the circumstances surrounding the injury, injury intent, socio-demographic details, use of health and social services in the 4 weeks prior to the injury and the EQ-5D (a measure of quality of life [16]) or the PedsQL (Quality of life for children (aged less than 16 years) [17] relating to the day before the injury. At 1 month, 4 months and 12 months post recruitment, participants will be asked to complete a questionnaire containing questions on whether they are still affected by their injury, use of health and social services and time off work in preceding 4 weeks, the EQ-5D/PedsQL relating to the day of questionnaire completion, the Work Limitations Questionnaire (self completed for adults) relating to the preceding 2 weeks and version 3 of the Health Utilities Index (HUI [18]) relating to the preceding 4 weeks. The choice of instruments was determined by a review of the literature and published guidelines. [19] All measures will be completed by the injured person or by proxy if necessary (except for the Work Limitations Questionnaire which can not be completed by proxy). Follow up questionnaires will be administered by post or participants will be able to complete a web-based questionnaire on a secure server with ID and password protection. Non-responders will be followed up by repeat mailed questionnaires and/or telephone reminders. Participants reporting that their injury no longer affects them will not be sent future follow-up questionnaires. A small incentive (£2 store voucher) will be sent with each follow up questionnaire. Also, all participants will be entered into a draw at the end of the study with 10 prizes of £100 of high street vouchers.

Data will be extracted from the medical records on date and time of injury, whether injury resulted from a road traffic accident, full text of diagnosis and treatment (including X-ray reports and surgical procedures), hospital admission and recommended follow-up. In the case of burns we will also record location, degree and percentage of body affected, in the case of head injury we will also record the lowest Glasgow Coma Score [20] and length of time of loss of consciousness. Socio-economic status will be based on area deprivation scores derived from the postcode of residence.

Where possible, the following information will be collected for patients who do not consent to the study: sex, age, place of injury and type of injury.

### Qualitative interviews

Semi-structured interviews will explore issues such as factors that facilitate or hinder recovery including access to health care and social support and issues surrounding the effects of insurance and compensation. Interviews will be conducted in the participant's own homes or by telephone and will be audio taped and transcribed. A total of 90 interviews will be conducted across 3 centres (Swansea, Bristol and Surrey). In each centre, 10 participants will be interviewed from each of three age groups; 5–24, 25–59 and 60 years and over. The sampling frame for the interviews will be stratified by centre, age and injury severity.

### Centres for recruitment

Recruitment will be undertaken in four geographic centres: Royal Surrey County Hospital in Surrey, Morriston Hospital in Swansea, Bristol Royal Infirmary in Bristol and the Nottingham University NHS Trust in Nottingham.

#### Ethical considerations

The study has multi-centre research ethics committee approval from the Dyfed Powys Local Ethics Committee (Number: 05/WMW01/23).

### Analysis

The analysis will provide estimates of the burden of injury using multiple approaches. Effects on disability and quality of life will assessed using the EQ-5D, the HUI, the Work Limitations Questionnaire, time off work and utilization of health and social care resources, by age (5–24, 25–59, 60+), gender, socio-economic status, injury setting (home, road, occupational, leisure), type of injury (burn, fracture, etc), anatomical site of injury and finally hospital admission status. Estimates will be made of the QALYs lost by combining the above variables with mortality data for the entire group and above subgroups. The distribution of recovery times will be calculated taking into consideration variables above (i.e injury type, age group etc). Where results are similar across injury types, sites or age groups the data will be combined to improve the precision of estimates. If we are able to successfully apply the ICISS to International Classification of Diseases (ICD) 10^th ^Edition codes from hospital admission data (Hospital Episode Statistics (HES) in England and Patient Episode Database for Wales (PEDW)), we will extrapolate from these data to produce national estimates of the burden of injury resulting from injuries requiring hospital admission based on the measures described above. We will also produce national estimates of the burden of injury associated with injuries requiring emergency department attendance based on the numbers and types of injuries attending hospital emergency departments, utilising regional surveillance systems where national data are not available, such as the All Wales Injury Surveillance System [AWISS] [21]. Derived estimates of post injury disability will be combined with published mortality data from the Office of National Statistics (ONS) to calculate the overall burden of injuries. We will carry out sensitivity analysis to explore possible effects of non-response bias. Qualitative interviews will be transcribed, analysed using thematic content analysis and inter-coder reliability will be assessed. Participants' experiences of life post injury will be described. Responses relating to utilisation of health and social services will be translated into resource implications using relevant published unit cost data, while the economic impact of work loss will be estimated using wage rates and measures of national output.

### Sample size

The sample size will be 1333, comprising 334 participants recruited from each centre over a 20 month period. This number was derived from the sampling frame in tables 1 and 3, aiming to recruit a minimum of approximately 15–20 participants in each cell in order that reasonably reliable estimates of the each of the measures can be obtained for each cell. The overall sample size and numbers within each injury category and group reflect pragmatic decisions based on available finances and a desire to cover as comprehensive a population of injury categories as possible.

In order to estimate the UK burden of injury it is necessary to extrapolate the findings from this study to all those attending similar emergency departments or admitted to hospital. Theoretically a large random sample of patients attending the emergency departments in each of the centres would be the ideal method to assess the overall burden of injury. However, because minor injuries are more common than moderate or severe injuries, this would result in large numbers of people with minor injuries being recruited and very few with moderate or severe injuries; which would result in very imprecise estimates of the burden of injury for moderate and severe injuries. Therefore, we undertook quota sampling to ensure a mix of different types of injury at different levels of severity. This method is likely to fail to recruit some uncommon injuries with a total sample of 1333. However, uncommon injuries are unlikely to make a large contribution to the estimates of the burden of injury at a national level. If data are deficient for certain important injuries we will explore the possibility of imputing data for national extrapolations from the most comparable study in the Netherlands using relative differences in quality of life in included and missing injury subtypes [6].

### Time scale

Participants will be recruited from September 2005 to April 2007 with follow-up completed in April 2008.

## Discussion

The study with a relatively large sample size, measurement of pre and post injury status and a potentially higher response rate than previous studies should constitute the most detailed and comprehensive study of injuries of varying severity to date. conducted to date in a population with a wide spread of injury severities. The methodological developments and data from this study should also make a substantial contribution to the international collaborative effort to more accurately assess the global burden of injuries.

In addition, this study will provide much improved estimates of the UK burden of injuries in terms of disability, cost and premature mortality. It is intended that this information will stimulate policy makers and practitioners to increase investment in effective injury prevention interventions and to support research into new interventions where the burden of injuries is high but evidence for effective interventions is lacking.

## Competing interests

The author(s) declare that they have no competing interests.

## Authors' contributions

RL, ET, DK, NC, SB, CP and CC wrote the original grant proposal. RC, LG, JS, AE, IP and FC contributed to amendments of the proposal in line with local implementation and best methods of recruitment and working in the emergency department and inpatient settings. All authors contributed to writing and approving this paper.

## Pre-publication history

The pre-publication history for this paper can be accessed here:


